# Dental Science

**Published:** 1889-05

**Authors:** 


### Bibliographical.

Dental Science: Questions and Answers on Dental Materia Medica, Dental Physiology, Dental Pathology and Therapeutics. By Luman C. Ingersoll, A. M., D. D. S., Dean of Dental Department of the State University of Iowa, 1882 to

1888. Second Edition. Publishers: Wilmington Dental M'f'g Co., Philadelphia, Pa. Price $2.00. Dr. Ingersoll in this second edition of his work has still further added to its value as an adjunct to the larger text-book. He remarks in the preface: "It is not intended that the text-book shall take the place of the lecture, but be used as the preacher uses the text of Scripture, as a basis of his sermon;" and he truly says: "the value of instruction does not depend upon the large amount presented to the mind of the pupil, but upon the amount grasped and held in memory."

In this progressive age when everything is conducted at such a rapid rate as would astonish our predecessors could they behold it, the general tendency on the part of the student is to accomplish a college course within the least time possible which necessarily implies a superficial knowledge, or one that is acquired by what is commonly designated as "cramming." Hence, a work of "questions and answers" may afford a temptation to the student to depend upon it to the exclusion of the regular text-book in which the subjects are discussed more exhaustingly, and the information imparted is more useful and comprehensive. Dr. Ingersoll has, however, by the manner in which he has arranged his work, avoided such an objectionable feature as that referred to, and presented a volume which must be of service to the student.

